# Key Considerations for Phase 2 or 3 Clinical Study Design of Anti-Inflammatory Agent for COVID-19 Treatment

**DOI:** 10.3389/fphar.2022.842836

**Published:** 2022-06-02

**Authors:** Yewon Park, Joo Young Na, Joo-Youn Cho, Jaeseong Oh, Su-jin Rhee

**Affiliations:** ^1^ Department of Clinical Pharmacology and Therapeutics, Seoul National University College of Medicine and Hospital, Seoul, South Korea; ^2^ Department of Pharmacy, Wonkwang University College of Pharmacy, Iksan, South Korea

**Keywords:** COVID-19, drug development, clinical trial design, anti-inflammatory agents, cytokine release syndrome

## Abstract

**Purpose:** Current understanding of COVID-19 disease progression suggests a major role for the “cytokine storm” as an important contributor to COVID-19 mortality. To prevent an exaggerated immune response and improve COVID-19 patient endpoints, anti-inflammatory therapeutics have been proposed as clinically useful in severe patients with COVID-19. The purpose of this study was to propose a clinical trial design for the development of anti-inflammatory agents for the treatment of COVID-19, taking into account the physiological and immunological process of COVID-19 and the treatment mechanism of anti-inflammatory agents.

**Methods:** We reviewed and analyzed the guidelines for the development of COVID-19 treatments and the treatment of COVID-19 by regulatory agencies and previously conducted clinical trials on anti-inflammatory drugs for COVID-19. Finally, after discussing with an advisory group, a synopsis was presented for an example protocol for a COVID-19 anti-inflammatory agent phase 2 or 3 study that considers the drug mechanism and the disease progression of COVID-19.

**Results:** A randomized, placebo-controlled, double-blind parallel-group design was suggested as a phase 2 or 3 trial design for developing an anti-inflammatory agent as a COVID-19 treatment. A key item of the example protocol specific to anti-inflammatory agents was the inclusion and exclusion criteria, taking into account the immunosuppressive effects of the drug, clinical time course of COVID-19 disease, and treatment guidelines for COVID-19. Time to recovery is the primary endpoint associated with clinical efficacy and is generally well accepted by many experts.

**Conclusion:** Through this suggested phase 2 or 3 study design of an anti-inflammatory drug for COVID-19, we provide a basis for a study design that can be utilized in clinical development by pharmaceutical companies which are developing a potential anti-inflammatory agent for COVID-19.

## 1 Introduction

Severe Acute Respiratory Syndrome Coronavirus 2 (SARS-CoV-2) is a highly contagious pathogenic coronavirus that emerged at the end of 2019, affecting the core of society and the economy beyond the health and public safety crisis. The impact of coronavirus disease of 2019 (COVID-19) is serious, extensive and detected worldwide and is still resurging after the spread of the delta variant which is more contagious than previous variants and a predominant variant of the virus in most parts of the world. The COVID-19 virus belongs to the beta-coronavirus and is mainly transmitted through close contact with respiratory droplets from a runny nose or cough within a radius of 2 m (Li H. et al., 2020; Setti et al., 2020). The incubation period of COVID-19 is 1–14 days (average 5–7 days), and the symptoms can range from fever, cough, difficulty breathing and pneumonia to critical disease including death due to severe respiratory failure caused by extensive lung damage (Li Q. et al., 2020; Yuki et al., 2020).

Cytokine storm syndrome (CSS) is an important clinical condition induced by cascades of cytokine activation, featuring overwhelming systemic inflammation, hemodynamic instability, and multiple organ failure (Gao et al., 2021). Patients with early signs of CSS in the early stages of infection (<5 days of symptoms) are known to be more likely to progress to the respiratory disease stage (Tang et al., 2020; Tay et al., 2020). In addition, the inflection point for disease with exacerbated respiratory symptoms is generally between 5–7 days of disease onset during which time targeted immunomodulatory therapy will be most beneficial in improving mortality and controlling excessive cytokine release (Buszko et al., 2020; Sinha and Calfee, 2021).

Treatments for COVID-19 can be classified into antiviral agents, convalescent plasma therapy, neutralizing antibodies and immunomodulators including anti-inflammatory and immune-boosting agents, which have different mechanisms of action and effects (Quek et al., 2021). Among these, anti-inflammatory agents may reduce the mortality in severe COVID-19 patients by suppressing the excessive immune response caused by COVID-19 infection (Boregowda et al., 2020; Sterne et al., 2020; Kalil et al., 2021). Several anti-inflammatory drugs including corticosteroids, interferons, cytokine inhibitors, and Janus kinase inhibitors (JAK inhibitors) have been studied for the treatment of COVID-19. Corticosteroids have immunomodulatory effects and are used as adjuvant treatment for acute respiratory distress syndrome (ARDS) and cytokine storm. Interleukin-6 (IL-6) receptor antagonists exhibit anti-inflammatory effects by reducing the elevation of IL-6 (Gordon et al., 2021; Horby et al., 2021; Veiga et al., 2021), and IL-6 is an important mediator for severe systemic inflammatory responses in patients with COVID-19. JAK inhibitors can suppress immune induction by interfering with the phosphorylation of signal transducer and activator of transcription (STAT), and several JAK inhibitors have been studied in COVID-19 patients (Burrage et al., 2020; Cao et al., 2020; Tufan et al., 2020; Kalil et al., 2021). Additionally, many potential anti-inflammatory drugs are under development and in the clinical stages (Patel et al., 2021).

Increasing the probability of clinical development success and accelerating drug development through the planning of well-designed clinical trials is one of the important drug development strategies to overcome the current COVID-19 pandemic situation. Many researchers and regulators have presented general considerations for the clinical trial design of vaccines or COVID-19 treatments through guidelines or review articles (Food and Drug Administration, 2020; Jiang et al., 2020; Zhang et al., 2020; Shi et al., 2021). However, more specific scientific rationales for the clinical study design of anti-inflammatory agents are needed for actual planning of clinical trials as different mechanisms of action, appropriate treatment timing, or target subjects may differ depending on the type of treatment. Therefore, the purpose of this study was to propose a clinical trial design specific to anti-inflammatory drugs by considering the therapeutic mechanism of anti-inflammatory drugs and the pathophysiology of COVID-19 and by analyzing the design and results of previous clinical trials for anti-inflammatory agents.

## 2 Materials and Methods

### 2.1 Literature Search

First, the clinical stages and management strategies for the clinical course of COVID-19 were investigated through a literature search. The pathological mechanisms related to the symptoms of COVID-19 disease and the pharmacological properties and mechanisms of anti-inflammatory drugs were investigated according to subcategories. Second, we reviewed domestic and international anti-inflammatory treatment strategy guidelines for COVID-19, including the “COVID-19 treatment guidelines” of the National Institutes of Health (NIH). Next, we reviewed the guidelines for developing a treatment for COVID-19, including the guidelines for “COVID-19: Developing drugs and biological products for treatment or prevention” of the U.S. Food and Drug Administration (FDA) and “Considerations in developing COVID-19 treatments” of the Ministry of Food and Drug Safety, 2020 (Table 1). Third, for consideration in the anti-inflammatory agent-specific clinical trial protocol, the clinical course of COVID-19, pharmacological properties of anti-inflammatory drugs, and domestic and foreign guidelines are summarized only as necessary.

**TABLE 1:** Lists of domestic and international guidelines for COVID-19 treatment and therapeutic development.

Guidelines for COVID-19 Treatment	The Korean Society of infectious diseases: Guidelines for the Korean Society of infectious diseases on COVID-19 medication Therapy
	WHO: Clinical Management of COVID-19
	NIH: COVID-19 Treatment guidelines
	EMA: Treatment and Vaccines for COVID-19
Guidelines for developing a treatment for COVID-19	Ministry of Food and Drug Safety: Considerations when developing a treatment for COVID-19
	U.S. FDA: COVID-19: Developing Drugs and Biological Products for Treatment or Prevention
	EMA: Guidance for medicine developers and other stakeholders on COVID-19
	NIH: ACTIV Update: Making Major Strides in COVID-19 Therapeutic Development

Abbreviations: COVID-19, coronavirus disease of 2019; WHO, World Health Organization; NIH, National Institutes of Health; EMA, European Medicine Agency; FDA, Food and Drug administration.

### 2.2 Analysis of Previous Clinical Studies

We searched the clinical trials of anti-inflammatory agents for COVID-19 within the clinical trial registry (clinicaltrials.gov) using the following keywords: “COVID-19” and “anti-inflammatory agent” until 21 April 2021. For the clinical trial of a COVID-19 anti-inflammatory agent conducted in domestic and abroad, major characteristics including study design, sample size, key primary endpoints, key secondary endpoints, and statistical methods were extracted and summarized. Based on this, common and specific contents were identified, and summarized to be reflected in the trial design. After discussing with an advisory group that included clinicians of the department of infectious diseases, we finally suggested a phase 2 or 3 study design of an anti-inflammatory agent for COVID-19 that considers the mechanism of anti-inflammatory agents and the immunological progress of COVID-19.

### 2.3 Proposal of an Anti-Inflammatory Agent-Specific Clinical Trial Design

The COVID-19 anti-inflammatory agent-specific clinical trial design section consists of common items from the guidelines for developing a COVID-19 treatment and from the previous clinical trial protocols for COVID-19 anti-inflammatory agents. The sections of the proposed clinical trial design included the study design, study population, efficacy endpoints, safety assessment, statistical considerations, and study procedure. Furthermore, the clinical course of COVID-19, the time of anti-inflammatory agent treatment, the mechanism of action, pharmacological characteristics of the COVID-19 anti-inflammatory agent and expert opinions were reflected in the above clinical trial design section as anti-inflammatory agent-specific contents. Detailed trial design contents were presented through the synopsis of the protocol.

## 3 Results

### 3.1 Summary of the Literature Search

Bhaskar, et al. reported that anti-inflammatory agents have a mechanism to block hyperinflammatory conditions by targeting cytokine-related signal transduction pathways and have pharmacological properties in which the risk of immunosuppression of anti-inflammatory agents may be greater than the risk of COVID-19 for immunocompromised patients (Bhaskar et al., 2020). In the literature related to the clinical course of COVID-19, Gentilotti et al. reported that it usually takes 5–7 days from the onset of symptoms of COVID-19 to hospitalization, which is an inflection point where respiratory symptoms are likely to worsen due to the action of excessive inflammation (Gentilotti et al., 2021).

For the recommended target population for the anti-inflammatory treatment, according to the NIH COVID-19 treatment guideline and EMA’s treatment and vaccine for COVID-19 guideline, anti-inflammatory agents are not generally recommended for non-hospitalized patients, and dexamethasone is typically recommended for hospitalized COVID-19 patients on oxygen or mechanical ventilation. Additional use of other anti-inflammatory agents, including baricitinib, is recommended to control the inflammatory response in patients whose disease worsens rapidly or is more severe. According to the COVID-19 medication therapy guidelines of the Korean Society of Infectious Diseases, steroid administration is recommended for severe or critical COVID-19 patients. Interleukin-6 (IL-6) inhibitors can be used for severe COVID-19 patients and are not recommended for mild COVID-19 patients.

For the clinical trial design, MFDS guideline (Considerations when developing a treatment for COVID-19) and FDA guideline (Developing Drugs and Biological Products for Treatment or Prevention) recommend a double-blind, randomized, placebo-controlled, and parallel group trial design to minimize bias in evaluating the efficacy and safety of the COVID-19 treatment. This placebo-control study should be conducted by adding a study drug or placebo to a standard therapy (according to clinical guidelines) considering the clinical status and severity of the recruited COVID-19 patients according to FDA guideline recommendation from an ethical point of view. The EMA guideline for medicine developers and other stakeholders on COVID-19 also recommends a large-scale, multicenter, multi-arm, randomized controlled clinical trial with a control arm without a test drug. NIH-funded “ACTIV-related trials” were randomized, placebo-controlled clinical trials. The analysis of the primary endpoint should be performed on all randomly assigned groups of subjects according to the MFDS guideline. The FDA guideline recommends a combined evaluation of clinical improvement and mortality at a specific time point as a clinical endpoint. For the selection of the sample size, there is no clear recommendation from the regulatory guidelines.

### 3.2 Results of the Analysis of the Previous Clinical Studies

Considering the results of previous anti-inflammatory agent trials for COVID-19 (NCT04381936, NCT04327388, NCT04401579, and NCT04421027), anti-inflammatory agents have been shown to be mainly effective in patients with severe or higher severity of COVID-19. The common design of the previous anti-inflammatory agent trials for COVID-19 was a randomized, placebo-controlled, parallel-group and multi-center trial. The key primary and secondary endpoints of the anti-inflammatory agent trials for COVID-19 were time to improvement, proportion of improvement, and all-cause mortality. In most of the anti-inflammatory agent trials, the number of subjects was a sample size for a statistical power of 75%–90% considering type 2 statistical errors. For statistical analysis, log-rank tests or Cox proportional risk models or Kaplan-Meier methods were used for survival analysis to evaluate variables of time to event such as recovery time (Table 2).

**TABLE 2:** Summary of previously conducted clinical trial designs of anti-inflammatory agents.

	Dexamethasone (NCT04381936)	Sarilumab (NCT04327388)	Baricitinib (NCT04401579)	Baricitinib (NCT04421027)
Study title	Randomized Evaluation of COVID-19 Therapy (RECOVERY Trial)	Evaluation of the Efficacy and Safety of Sarilumab in Hospitalized Patients With severe or critical COVID-19	A Multicenter, Adaptive, Randomized Blinded Controlled Trial of the Safety and Efficacy of Investigational Therapeutics for the Treatment of COVID-19 in Hospitalized Adults (ACTT-2)	A Randomized, Double-Blind, Placebo-Controlled, Parallel-Group Phase 3 Study of Baricitinib in Patients With COVID-19 Infection (COV-BARRIER)
Objective	To provide reliable evidence on the efficacy of candidate therapies for confirmed COVID-19 infection in hospitalized adult patients receiving usual standard of care	To evaluate the clinical efficacy of sarilumab relative to the control arm in adult patients hospitalized with severe or critical COVID-19	To evaluate baricitinib plus remdesivir in hospitalized adults with COVID-19	To evaluate the efficacy and safety of baricitinib in combination with standard of care for the treatment of hospitalized adults with COVID-19
Study design	Randomized, controlled, open-label phase 3 trial	Adaptive phase 3, randomized, double-blind, placebo-controlled trial	Double-blind, randomized, placebo-controlled phase 3 trial	Double-blind, randomized, placebo-controlled phase 3 trial
Sample size	45,000 participants	420 participants	1033 participants	1585 participants
Key primary endpoint	Day 28 all-cause mortality	Time to improvement in clinical status of participants using 7-point ordinal scale score	Time to recovery during the 28 days using the eight-category ordinal scale	Proportion who progressed to high-flow oxygen, non-invasive ventilation, invasive mechanical ventilation, or death by Day 28
Key secondary endpoint	Duration of hospital stay over the Day 28 period Composite endpoint of death or need for mechanical ventilation or ECMO over the Day 28 period	Proportion of patients alive at Day 29	Clinical status at Day 15, based on the eight-category ordinal scale	All-cause mortality by Day 28
Statistical methods	Sample sizes: not be estimated All-cause mortality: Hazard ratio from Cox regression Cumulative mortality over the 28-day period: Kaplan–Meier survival curves Duration of hospital stay over the Day 28 period and the endpoint of successful cessation of invasive mechanical ventilation: Cox regression	Sample size: 90% or greater power for pairwise comparison Time to improvement in Clinical Status of Participants: Stratified log-rank test with treatment as a fixed factor Estimation of treatment effect: Hazard ratio (HR) generated using a stratified Cox proportional hazards model The proportion of patients alive at Day 29: Cochran-Mantel-Haenszel test	Sample size: 85% power, a two-sided type I error rate of 5% Time to Recovery during the 28 days: Log-rank test Clinical status at Day 15, based on the eight-category ordinal scale: Single primary hypothesis test (no adjustments for multiplicity)	Sample size: 75% of the total α Proportion who progressed to high-flow oxygen, non-invasive ventilation, invasive mechanical ventilation, or death by Day 28: Odds ratios (ORs) from Logistic regression models (multiple imputation method) ll-cause mortality by Day 28: Hazard ratios (HRs) from Cox proportional hazard models

Abbreviations: COVID-19, coronavirus disease of 2019; ECMO, extracorporeal membrane oxygenation.

### 3.3 Suggested Study Design for an Anti-inflammatory Agent-Specific Clinical Trial

#### 3.3.1 Study Design

**FIGURE 1:**
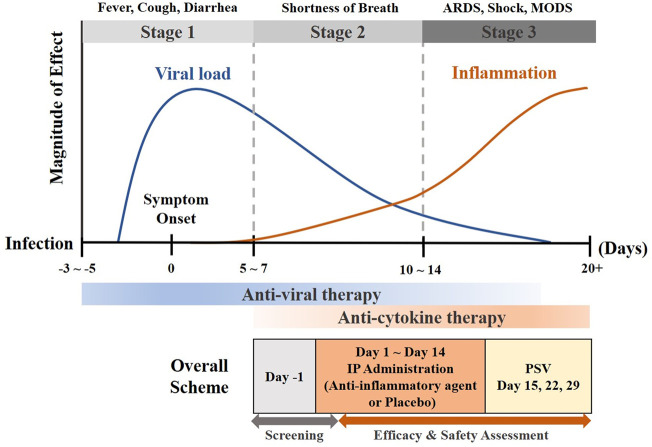
Clinical time course of COVID-19 and study design (adapted from (Aguilar et al., 2020)) Abbreviations: ARDS, acute respiratory distress syndrome; COVID-19, coronavirus disease of 2019; IP, investigational product; MODS, multiple organ dysfunction syndrome; PSV, post-study visit.

A randomized, placebo-controlled, double-blind parallel-group design was suggested as a phase 2 or 3 trial design for developing an anti-inflammatory agent for COVID-19. The main sections of the anti-inflammatory agent-specific protocol were the inclusion and exclusion criteria, considering the immunosuppressive effect of the drug, the clinical time course of the COVID-19 disease, and the treatment guidelines for COVID-19. The detailed clinical trial design was presented through a synopsis of the anti-inflammatory agent-specific clinical trial protocol (Figure 1, Supplementary Tables S1, S2).

#### 3.3.2 Study Population

The major target population was hospitalized patients with severe or higher severity of COVID-19, and immunocompromised patients were presented as the main exclusion targets (Tables 3, 4). Considering the COVID-19 severity categorization of the FDA guideline, patients with shortness of breath or dyspnea at rest, saturation (SpO2) less than 94%, and requiring supplemental oxygen therapy, mechanical ventilation, or extracorporeal membrane oxygenation (ECMO) may be included in the severe or higher severity category (Table 3).

**TABLE 3:** Examples of inclusion criteria for the study design of an anti-inflammatory agent for COVID-19.

1 Adults ≥19 years of age at time of screening
2 Subject admitted to a hospital with SARS-CoV-2 infection confirmed by RT-PCR
PCR positive in sample collected <72 h prior to randomization
3 Subject who can be classified as severe or higher in the COVID-19 severity category with one or more of the following conditions
Severe systemic symptoms such as shortness of breath or difficulty breathing during rest, and respiratory rate ≥30 times/minute
SpO2 <94% or PaO2/FiO2 on room air
Requiring supplemental oxygen
Requiring mechanical ventilation or ECMO
Lung infiltrates confirmed by imaging findings >50%
4 Female subject who is neither pregnant nor lactated or surgically infertile status (bilateral tubal occlusion, hysterectomy, bilateral ovarian resection, etc.)
5 Subject who agrees to not participate in other clinical trial for the treatment of COVID-19 during the study
6 Subject who voluntarily decides to participate and agrees to abide by the precautions with written consent after receiving a sufficient explanation and fully understanding of this study comply with all the protocol requirements by signing informed consent form after being informed of the nature of this study and fully understanding this study
7 Subjects who were deemed as eligible subjects by investigators on their physical examination, laboratory findings, and medical examination by interview

Abbreviations: COVID-19, coronavirus disease of 2019; RT-PCR, real-time polymerase chain reaction; ECMO, extracorporeal membrane oxygenation.

**TABLE 4:** Examples of exclusion criteria for a study design of an anti-inflammatory agent for COVID-19.

1 Subject who has hypersensitivity to the drug containing components of the study drug class or other drugs, or has a history of clinically significant allergic reactions
2 Subject with other bacterial, fungal, viral or other infections excluding SARS-COV-2 infection at the time of screening
3 Anticipated discharged from the hospital or transfer to a hospital where research cannot be conducted within 72 h
4 Subject who shows the following results in the screening test
ALT or AST >5 times the upper limit of normal
eGFR <30 ml/min
ANC <1000 cells/microliter
ALC <2000 cells/microliter
Subjects who show a positive result for a serology test (HBsAg, Anti-HCV, HIV Ab, or VDRL)
5 Subject who has a history of receiving either convalescent plasma or intravenous immunoglobulin for COVID-19
6 Received other immunosuppressants in the 4 weeks prior to screening and in the judgement of the investigator, the risk of immunosuppression with the study drug is larger than the risk of COVID-19
7 Has received any live vaccine (that is, live attenuated) within 4 weeks before screening, or intends to receive a live vaccine (or live attenuated) during the study
8 Subject who is considered to be ineligible for participation in this study by the investigator’s discretion based on laboratory results and other reasons

Abbreviations: ALC, absolute lymphocyte count; ALT, alanine transaminase; ANC, absolute neutrophil count; Anti-HCV, hepatitis C virus antibody; AST, aspartate aminotransferase; COVID-19, coronavirus disease of 2019; eGFR, estimated glomerular filtration rate; HBsAg, hepatitis B surface antigen; HIV Ab, human immunodeficiency virus antibody; VDRL, venereal disease research laboratory.

#### 3.3.3 Efficacy Assessment

The primary objective of this phase 2 or 3 trial is to evaluate the efficacy of an anti-inflammatory agent for COVID-19 against a standard treatment in patients with a COVID-19 infection. The time to recovery was suggested as a primary endpoint which is related to the clinical efficacy and generally well accepted by many experts. The definition of time to recovery is the first day on which the subject satisfies one of the 1 to 3 categories from the 8-category ordinal scale used in ACTT-2. The secondary objective of this study is to evaluate the safety of an anti-inflammatory agent in patients with a COVID-19 infection. The secondary endpoint can be considered as the clinical status evaluated on a clinical ordinal scale, the oxygen treatment period, the duration of the hospitalization, and the mortality at each point in time (Table 5).

**TABLE 5:** Suggested study endpoint of the study design of an anti-inflammatory agent for COVID-19.

Primary endpoint
Time to recovery (day): The first day on which the subject satisfies one of the 1–3 categories from the following 8-category ordinal scale
Rate of invasive mechanical ventilation or all-cause mortality by Day 29
*8-category ordinal scale
1 Not hospitalized, no limitations on activities
2 Not hospitalized, limitation on activities and/or requiring home oxygen
3 Hospitalized, not requiring supplemental oxygen—no longer requires ongoing medical care
4 Hospitalized, not requiring supplemental oxygen—requiring ongoing COVID-19 related medical care
5 Hospitalized, requiring supplemental oxygen
6 Hospitalized, on non-invasive ventilation or high flow oxygen devices
7 Hospitalized, on invasive ventilation or extracorporeal membrane oxygenation)
8 Death
Secondary endpoint
Subject’s clinical status assessed using the 8-category ordinal scale at Day 15
Time to an improvement in each of 1 and 2 categories from Day 1 (baseline) using the 8-category ordinal scale
AuthorAnonymous, 11 - Mean change in the 8-category ordinal scale from Day 1 (baseline) to Day 3, 5, 8, 11, 15, 22 and 29
Time to discharge or to a NEWS of ≤2 and maintained for 24 h, whichever occurs first
AuthorAnonymous, - Mean change from Day 1 (baseline) to Days 3, 5, 8, 11, 15, and 29 in NEWS
Days of oxygenation (supplement oxygen, noninvasive ventilation or high-flow oxygen) use
up to Day 29
Duration of hospitalization (days)
Day 14 and Day 28 mortality
Safety assessment
Physical examination
Clinical laboratory tests
Vital signs
12 Lead ECG and chest X-ray test
Adverse event monitoring

Abbreviations: COVID-19, coronavirus disease of 2019; NEWS, National early Warning Score; ECG, electrocardiogram.

#### 3.3.4 Safety Assessment

Adverse events occurring after the administration of a study drug can be assessed by aggregating the results of the clinical laboratory tests, vital signs, and physical examination conducted at the institution of the clinic trial (Table 5). Clinical laboratory tests may include tests to check health conditions, blood clotting tests and a virological assessment associated with the administration of an anti-inflammatory agent for COVID-19. Adverse events can be classified as adverse drug reactions, serious adverse events/adverse reaction, and unexpected adverse reactions according to each definition and should be standardized and collected according to the Medical Dictionary for Regulatory Activities. The severity of the adverse events should be determined by the investigator based on clinical judgment according to objective severity-related classification criteria. In addition, all adverse reactions should be assessed for causality with the study drug and actions taken in relation to adverse reactions should be recorded.

#### 3.3.5 Statistical Considerations

The appropriate number of subjects can be the sample size for a statistical power of 85% or 90% taking into account type 2 statistical errors. For example, in a previous study using baricitinib as a treatment for COVID-19 (Kalil et al., 2021), the time to recovery was statistically significantly faster in the baricitinib group compared to the placebo group, and the odds ratio (95% confidence interval) for the treatment and was 1.16 (1.01–1.32). Based on the study results, when the relative hazard is set to 1.16 and the statistical power is set to 80%, the appropriate number of subjects considered necessary is 675 patients per treatment group and a total of 1,350 or more patients.

Statistical analysis can be conducted according to the characteristics of the evaluation variables. For the endpoint time to event as a primary endpoint, the Log-rank test or Cox proportional-hazard model or Kaplan-Meier method can be used for survival analysis. The test drug should show superiority over the placebo by a two-sided test at a significance level of 0.05. For dichotomous endpoints, logistic regression can be used, and a proportional probabilistic model can be used for ordinal endpoints. An analysis of variance (ANOVA) model can be used for continuous endpoints, and a mixed-effects model of repeated measures can be used to evaluate the continuous endpoints over time. For some endpoints, the statistical model can be adjusted for treatment and baseline stratification factors.

#### 3.3.6 Study Procedure

Screening tests such as medical examination by interview, physical examination, clinical laboratory test, etc. can be conducted on Day -1 or Day 1 for COVID-19 patients who voluntarily agree to participate in this study to select test subjects deemed suitable for this clinical trial. The subjects determined to be eligible for this trial are stratified by SARS-CoV-2 vaccination, clinical trial institutions, and disease severity at the time of randomization and randomly assigned to one of two treatment groups on Day 1. Subjects can be administered an anti-inflammatory agent or placebo assigned on Day 1 to Day 14. The efficacy and safety/tolerability assessment can be carried out according to the specified performance schedule. The researcher can conduct examinations through daily interviews with all the subjects during the hospitalization period, and when discharged from the hospital during the study period, the safety assessments can be performed at Day 15, 22, and 29, respectively. Other drugs used prior to participation in the study should be discontinued during clinical trials, and no separate washout period is required for discontinuation of other drugs. However, standard care that is being used on the basis of regulatory guidelines or other recommendations for the treatment of COVID-19 can be maintained during clinical trials, and standard treatment drugs should be specified in clinical study reports.

## 4 Discussion

Zhang et al. (Zhang et al., 2020) and Jiang et al. (Jiang et al., 2020) presented considerations for developing COVID-19 vaccines in terms of clinical trial design and the analysis results of the characteristics of clinical trial design for COVID-19 treatments registered in the International Clinical Trials Registry Platform. Shi et al. (Shi et al., 2021) suggested that barriers with previous clinical trials have been attributed to a lack of pathological understanding of the disease, unoptimized doses, poorly defined treatment timing windows, nonspecific endpoint measurements, and nonrandomized and underpowered trial designs. To address these challenges, the authors suggested that well-designed clinical trials need to be planned by following translational science principles with knowledge on the COVID-19 pathology and the dynamics of the immune response for COVID-19 and implementing 4R (appropriate patient, right drug, right dosage, and right timing) concepts to increase test success and generate high-quality data. In this study, we designed an example study for anti-inflammatory drugs in hospitalized patients with severe or a higher severity of COVID-19 by considering the pharmacological mechanisms of anti-inflammatory drugs and the immunological processes of COVID-19.

For the target population of this study design, we judged that inpatients would be the most appropriate given that it usually takes between 5 and 7 days from symptom onset to hospitalization for COVID-19 which is an inflection point for respiratory symptoms to likely worsen as a function of excessive inflammation (Gentilotti et al., 2021). Considering the timing of the anti-inflammatory treatment and the results of anti-inflammatory agent trials for COVID-19, it seemed appropriate to proceed with clinical trials of anti-inflammatory drugs for COVID-19 for hospitalized patients with severe or a higher severity of COVID-19. For small-scale proof-of-concept (POC) trials to identify test drugs, patients with a certain severity of COVID-19 infection can be considered for who the treatment is expected to be most effective depending on the type of anti-inflammatory agent and its mechanism of action. For confirmatory clinical trials in which it is important to create a reliable safety and efficacy database, it is considered appropriate to allocate equally patients to the test drug and placebo groups through a stratified randomization without an inclusion criterion for selecting patients with a specific severity. Those who have been administered convalescent plasma therapy or intravenous immunoglobulin (IVIg) to treat COVID-19 should be excluded because it may affect the results of this study. To minimize the risk of immunosuppression, it is also recommended to exclude those with live vaccines within 4 weeks of participation in clinical trials. But it is considered appropriate to ensure that COVID-19 vaccinated patients are evenly assigned to study drugs and placebo through stratified randomization without being excluded because the COVID-19 vaccine is not a live vaccine (Table 4).

The effectiveness of the study drug for COVID-19 should be evaluated in comparison with a placebo or active control in terms of the clinical implications of disease in the development of COVID-19 treatments. The interpretation of the efficacy endpoint of an anti-inflammatory agent for COVID-19 and time frame setting can vary depending on the characteristic of the treatment, study population and the severity of COVID-19. For the safety assessment of this study design, a virological assessment (viral titer, CT value and time to viral negative) is considered more appropriate for the safety evaluation or discharge criteria than an efficacy endpoint because no clear relationship has been established between an efficacy endpoint and clinical symptoms along with a benefit.

As mentioned in Felsenstein and Reiff, 2021, the inflammatory process changes pathophysiologically as COVID-19 progresses. Therefore, it is important to predict disease progression of COVID-19 through appropriate biomarkers or inflammatory markers; these markers should be considered in patient risk stratification and drug efficacy assessment with respect to disease progression. In addition, by using specific markers for each stage of COVID-19 progression, the timing of anti-inflammatory treatment might be adjusted in consideration of the target specificity of the drug and the severity and inflammatory process of the disease. For example, in hospitalized patients who do not require supplemental oxygen therapy (e.g., WHO stage 3), Angiopoietin 2 and circulating endothelial cells may be useful biomarkers for the assessment of disease progression before oxygen requirement occur. On the other hand, biomarkers for the formation of neutrophil extracellular traps (i.e., NETosis) associated with the bacterial and viral defense system of neutrophils are significantly elevated in hospitalized patient requiring non-invasive ventilation or high-flow oxygen (e.g., WHO stage 5). Also, secondary fungal infections (e.g., mucormycosis, aspergillus) should be carefully monitored during follow-up visits in long-term treated patients or who received long half-life anti-inflammatory agents, and safety monitoring plan for secondary infections should be provided during the clinical trial of anti-inflammatory agents (Felsenstein and Reiff, 2021).

Generally, in the clinical stage, a drug is developed as a treatment by exploring its safety, tolerability and pharmacokinetic characteristics in humans through a phase 1 clinical trial; the therapeutic efficacy and dosage-response relationships are explored in a phase 2 clinical trial, and the clinical efficacy is confirmed through phase 3 clinical trials. To successfully achieve the objective of a phase 2 or 3 clinical study for exploring or confirming the therapeutic efficacy of anti-inflammatory treatment for COVID-19, it might be necessary to consider the stratified randomization of severity in patients from severe to a higher severity of COVID-19 for accurate and reliable assessments of safety and efficacy.

Anti-inflammatory drug development clinical trials for COVID-19 were investigated until April 2021, and since then, there have not been many cases of development of anti-inflammatory drugs among COVID-19 treatments, and only limited data exist to confirm the specific design of actual clinical trials. Thus, we focused on guidelines for general considerations in the development of a COVID-19 treatment. Therefore, there is a limitation in that this study does not reflect the latest clinical trial results and clinical development status reported in the second half of 2021. Furthermore, because this study design is intended for repurposing or finding novel small molecule anti-inflammatory agents, additional assessments including immunogenicity assessment, and monitoring of hypersensitivity reactions and infusion-related reactions are necessary for biologic anti-inflammatory agents. Therefore, it is recommended to appropriately modify and use these contents according to the specific characteristics and subcategory of the study drug. In addition, at this point, COVID-19 disease is spreading the more contagious delta and omicron variants of the virus than previous strains, and the status of treatment development continues to change, and additional considerations may be needed for this situation. It should be noted that some content (e.g., clinical trials for inpatients) can be selectively applied in other countries because social policies for COVID-19 response vary from country to country and that the proposal of this study considered the situation in Korea. Moreover, it should be noted that this is only a methodological suggestion that does not take into account the specific environment and guidelines of the hospital, and the patient’s medical history or the difference in treatment administered according to the characteristics of the patient. However, through the methodological proposals in this paper, clear and precise information on the effects of anti-inflammatory drugs can be obtained. Nevertheless, to the best of our knowledge, this paper is meaningful in that it is the first article presented on key considerations of study design for anti-inflammatory agents for COVID-19 considering the mechanism of anti-inflammatory drugs and the timing of the COVID-19 immunology. In this paper, we reiterate the importance of well-designed clinical trials to increase the chances of success in developing COVID-19 anti-inflammatory drugs and accelerate clinical development. We designed an example study of anti-inflammatory treatments in hospitalized patients with severe or a high severity of COVID-19, taking into account the pharmacological mechanism of anti-inflammatory agents and the immunological process of COVID-19. The clinical trial design considerations proposed in this study, reflecting knowledge of the dynamics of immune responses to COVID-19 and the mechanism of drugs, can be used to plan well-designed clinical trials to enhance development success and generate high-quality data.

## 5 Conclusion

This phase 2 or 3 study design of an anti-inflammatory drug for COVID-19 was established considering the mechanism of drugs and the disease progress of COVID-19, and it can be useful for drug developers developing an anti-inflammatory agent for COVID-19.

## Data Availability

The original contributions presented in the study are included in the article/Supplementary Material, further inquiries can be directed to the corresponding authors.
