# Supplementary material for: Key Considerations for Phase 2 or 3 Clinical Study Design of Anti-Inflammatory Agent for COVID-19 Treatment
Source: Front Pharmacol. 2022 Jun 2;13:842836. doi: 10.3389/fphar.2022.842836 (PMC9201470; doi:10.3389/fphar.2022.842836)
Supplement: Supplementary file 1 [file Table1.DOCX]

**Supplementary Table S1**. Example of a phase 2 or 3 anti-inflammatory specific protocol synopsis

| Title of Study | A Randomized, Placebo-Controlled, Double-Blind Clinical Study to Evaluate the Efficacy and Safety of Drug X in Patients with COVID-19 |
| --- | --- |
| Phase of Study | Phase 2 or 3 |
| Principal Investigator | <To be determined later> |
| Sponsor | <To be determined later> |
| Study Institution | <To be determined later> |
| Analysis Institution | <To be determined later> |
| Study Period | 12 months from the date of IND or IRB approval |
| Indication | Coronavirus disease of 2019 (COVID-19) |
| Objectives | - Primary objective: To evaluate the effectiveness of Drug X compared to standard treatment in patients with COVID-19 - Secondary objective: To evaluate the safety of Drug X in patients with COVID-19 |
| Investigational Product | Test drug: Drug X |
| Number of Subjects | - 0000 subjects - Basis of the number of subjects: The sample size for 85% or 90% statistical power was calculated considering type II statistical errors.   *Example: In a previous study using baricitinib as a treatment for COVID-19 (Kalil AC et al., N Engl J Med. 2020 Dec), the time to recovery was statistically significantly faster in the baricitinib group compared to the placebo group, and the odds ratio for treatment and 95% confidence interval was 1.16 (1.01–1.32). Based on the study results, when the relative hazard was set to 1.16 and the statistical power was set to 80%, it was calculated that 675 patients per treatment group would be needed, and a total of more than 1350 patients were calculated as required for the study.* |
| Criteria for Inclusion and Exclusion | - **Inclusion Criteria**  1. Adults aged ≥ 19 years at screening 2. Subject admitted to a hospital with SARS-CoV-2 infection confirmed by RT-PCR    - PCR positive in sample collected < 72 hours prior to randomization 3. Subject who can be classified as severe or higher in the COVID-19 severity category with one or more of the following conditions:  - Severe systemic symptoms such as shortness of breath or difficulty breathing during rest, and respiratory rate ≥ 30 times/minute - SpO_2_ < 94% or PaO_2_ / FiO_2_ on room air - Requiring supplemental oxygen - Requiring mechanical ventilation or Extracorporeal membrane oxygenation (ECMO) - Lung infiltrates confirmed by imaging findings > 50%  1. Female subject who is neither pregnant nor lactated or surgically infertile status (bilateral tubal occlusion, hysterectomy, bilateral ovarian resection, etc.) 2. Subject who agrees to not participate in other clinical trial for the treatment of COVID-19 during the study 3. Subject who voluntarily decides to participate and agrees to abide by the precautions with written consent after receiving a sufficient explanation and fully understanding of this study comply with all the protocol requirements by signing informed consent form after being informed of the nature of this study and fully understanding this study 4. Subjects who were deemed as eligible subjects by investigators on their physical examination, laboratory findings, and medical examination by interview  - **Exclusion Criteria**  1. Subject who has hypersensitivity to the drug containing components of the drug X class or other drugs, or has a history of clinically significant allergic reactions 2. Subject with other bacterial, fungal, viral or other infections excluding SARS-COV-2 infection at the time of screening 3. Anticipated discharged from the hospital or transfer to a hospital where research cannot be conducted within 72 hours 4. Subject who shows the following results in the screening test  - ALT or AST >5 times the upper limit of normal - eGFR <30 ml/min - ANC <1000 cells/microliter - ALC <2000 cells/microliter - Subjects who show a positive result for a serology test (HBsAg, Anti-HCV, HIV Ab, or VDRL)  1. Subject who has a history of receiving either convalescent plasma or intravenous immunoglobulin for COVID-19 2. Received other immunosuppressants in the 4 weeks prior to screening and in the judgement of the investigator, the risk of immunosuppression with the study drug is larger than the risk of COVID-19 3. Has received any live vaccine (that is, live attenuated) within 4 weeks before screening, or intends to receive a live vaccine (or live attenuated) during the study 4. Subject who is considered to be ineligible for participation in this study by the investigator’s discretion based on laboratory results and other reasons |
| Study procedure | A randomized, double-blind, placebo-controlled, parallel group study is performed. Screening tests such as interviews, physical examinations, and clinical tests are conducted on Day -1 or Day 1 for COVID-19 patients who have voluntarily expressed their intention to participate in this study. Subjects deemed suitable for this clinical trial are selected. Subjects are administered the assigned test drug or placebo in the morning of Day 1 to Day 14. Evaluation of efficacy, safety/tolerability is conducted according to the planned clinical trial schedule. The researcher interviews all subjects daily during the hospitalization period, and subjects discharged during the study period visit each of Day 15, Day 22, and Day 29, respectively, to perform the safety evaluation. If it is difficult for the subject to visit the study institution for other reasons, including infection, the subject can be interviewed by phone.  Other drugs used prior to participation in this study should be discontinued during the study period and no separate washout period due to discontinuation of other drugs is required. However, standard treatments that were being used based on regulatory guidelines or other recommendations for the treatment of COVID-19 infection can be maintained during the study period. These preceding and combined drugs related to these standard treatments should be specified in the clinical study report.   - **Screening (Day -1 or Day 1)**   For volunteers, screening tests such as questionnaires, physical examinations, and clinical tests are conducted at Day -1d or Day 1, and subjects judged to be suitable for this clinical trial are selected.   - **Treatment period (Day 1~Day 14)**   Subjects judged to be suitable for this clinical trial are stratified according to the following and randomly assigned to one of two treatment groups (test drug group or placebo group) on Day 1.   - SARS-CoV-2 vaccination - Clinical trials institution - Severity of COVID-19 disease at the time of randomization   - Less severe disease: Baseline (1d) 8-Ordinal scale category 5   - Severe disease: Baseline (1d) 8-Ordinal scale category 6 or 7   Subjects receive the assigned test drug or placebo in the morning of Day 1 - Day 14. Efficacy, safety/tolerability evaluation is carried out according to the planned study schedule.   - **Discharge (Day 14) and Follow-up visits (Day 15, Day 22, Day 29)**   Subjects complete the administration of investigational drugs by Day 14 or until discharge, and visit Day 15, Day 22, and Day 29 for follow-up and perform the prescribed schedule. Subjects who have dropped out of the clinical trial should visit within 7 days from the last day of the investigational drug administration or the date of decision to drop out to conduct the prescribed schedule. |
| Assessment methods | - **Efficacy endpoints** - **Primary endpoint**  1. Time to recovery (day): The first day on which the subject satisfies one of the 1 ~ 3 categories from the following 8-category ordinal scale 2. Rate of invasive mechanical ventilation or all-cause mortality by Day 29   **▶ 8-category ordinal scale**   - 1. Not hospitalized, no limitations on activities   2. Not hospitalized, limitation on activities and/or requiring home oxygen   3. Hospitalized, not requiring supplemental oxygen – no longer requires ongoing medical care   4. Hospitalized, not requiring supplemental oxygen – requiring ongoing COVID-19 related medical care   5. Hospitalized, requiring supplemental oxygen   6. Hospitalized, on non-invasive ventilation or high flow oxygen devices   7. Hospitalized, on invasive ventilation or extracorporeal membrane oxygenation   8. Death - **Secondary endpoint**  1. Subject’s clinical status assessed using the 8-category ordinal scale at Day 15 2. Time to an improvement in each of 1 and 2 categories from Day 1 (baseline) using the 8-category ordinal scale 3. Mean change in the 8-category ordinal scale from Day 1 (baseline) to Day 3, 5, 8, 11, 15, 22 and 29 4. Time to discharge or to a National Early Warning Score (NEWS) of ≤ 2 and maintained for 24 hours, whichever occurs first 5. Mean change from Day 1(baseline) to Days 3, 5, 8, 11, 15, and 29 in NEWS 6. Days of oxygenation (supplement oxygen, noninvasive ventilation or high-flow oxygen) use up to Day 29   ▶ National Early Warning Score   \| Physiological  parameter \| Score \| \| \| \| \| \| \| \| --- \| --- \| --- \| --- \| --- \| --- \| --- \| --- \| \| 3 \| 2 \| 1 \| 0 \| 1 \| 2 \| 3 \| \| Respiration rate (per minute) \| ≤8 \|  \| 9-11 \| 12-20 \|  \| 21-24 \| ≥25 \| \| SpO_2_ Scale 1 (%) \| ≤91 \| 92-93 \| 94-95 \| ≥96 \|  \|  \|  \| \| SpO_2_ Scale 2 (%) \| ≤83 \| 84-85 \| 86-87 \| 88-92  ≥93 on air \| 93-94 on oxygen \| 95-96 on oxygen \| ≥97 on oxygen \| \| Air or oxygen? \|  \| Oxygen \|  \| Air \|  \|  \|  \| \| Systolic blood pressure (mmHg) \| ≤90 \| 91-100 \| 101-110 \| 111-219 \|  \|  \| ≥220 \| \| Pulse (per minute) \| ≤40 \|  \| 41-50 \| 51-90 \| 91-110 \| 111-130 \| ≥131 \| \| Consciousness^*^ \|  \|  \|  \| Alert \|  \|  \| VPU \| \| Temperature (℃) \| ≤35.0 \|  \| 35.1-36.0 \| 36.1-38.0 \| 38.1-39.0 \| ≥39.1 \|  \|   * Alert: The patient is awake/ V: The patient responds to a verbal stimulus/ P: The patient responds to a pain stimulus/ U: Unresponsive   - **Safety assessment**  1. Physical examination 2. Clinical laboratory tests 3. Vital signs 4. 12 Lead ECG and chest X-ray test |
| Statistical methods | - **General statistical analysis**   This study is a placebo-controlled randomized study to show superiority, and a two-sided test is performed under a significance level of 0.05. Each data point is summarized and presented with appropriate descriptive statistics according to its characteristics (e.g., categorical variables are presented as percentages; continuous variables are presented as the mean and 95% confidence intervals, and time to event variables are presented as medians) The time to event data are presented as the Kaplan-Meier survival curve and its 95% confidence interval.   - **Descriptive statistical analysis**   Descriptive statistics (mean, standard deviation, median value, minimum value, maximum value, etc.) or frequency, ratio of categories, etc. are presented according to the characteristics of the data for demographic information such as age, height, and weight for all subjects given random assignment numbers (Intention-To-Treat population).   - **Efficacy statistical analysis**   The efficacy data are included for all subjects (Intention-To-Treat) assigned random assignment numbers.  The primary efficacy evaluation variable, "Time to recovery after administration of the investigational products," is presented in stratification according to the presence or absence of COVID-19 vaccination and the severity at the time of random assignment, and statistical significance is tested through log-rank tests. The event of death is judged as a case of not recovering and is evaluated as being censored on Day 29.  Among the secondary efficacy evaluation variables, the clinical status of patients evaluated by the 8-ordinal scale at Day 15 is analyzed through a proportional odds model. The treatment group, vaccination status, and severity at the time of randomization are analyzed by including the proportional odds model as covariates, and the odds ratio and *p*-value according to the treatment group are presented. In addition, the 95% confidence interval of the number, ratio, and odds ratio of subjects for each scale at Day 15 for each treatment group is presented.  For the time at which the clinical ordinal scale (8-ordinal scale) is improved, the time at which the National Early Warning Score is improved, and the mortality rate at Day 14 and Day 28, log-rank test results are presented using Cox's proportional hazards model. The mean amount of change in the category at each time point, the mean amount of change in the National Early Warning Score, the hospitalization period, and the number of days receiving oxygen therapy are presented as descriptive statistics.   - **Safety statistical analysis**   The safety evaluation data are included for all subjects administered the investigational products at least once.  Adverse events are coded using Medical Dictionary for Regulatory Activities (MedDRA), and the system organ class, the duration of adverse events, the number of occurrences, the number of subjects to be tested, severity, seriousness, and causal relationships with investigational products are summarized using descriptive statistics for each treatment group. For deaths or composite endpoints, a method for analyzing time to event data can be applied. Serious reactions or adverse events that caused suspension of participation in the study are summarized and presented in a separate table. Differences in the incidence of adverse events between treatment groups can be compared using appropriate parametric/nonparametric statistical tests such as Chi-square test and Fisher's effect test. In addition, clinically significant results of vital signs and clinical laboratory test results are stratified into severity to present the analysis results. |

**Abbreviations**: ALC, absolute lymphocyte count; ALT, alanine transaminase; ANC, absolute neutrophil count; Anti-HCV, hepatitis C virus antibody; AST, aspartate aminotransferase; COVID-19, coronavirus disease of 2019; eGFR, estimated glomerular filtration rate; HBsAg, hepatitis B surface antigen; HIV Ab, human immunodeficiency virus antibody; VDRL, venereal disease research laboratory.

**Supplementary Table S2**. **E**xample of a phase 2 or 3 anti-inflammatory specific overall study plan

|  | Screening Period | Treatment Period | | | | | | | | | | | Follow-up visits | | |
| --- | --- | --- | --- | --- | --- | --- | --- | --- | --- | --- | --- | --- | --- | --- | --- |
|  | Day -1 or Day 1 | Day 1 | Day 2 | Day 3 | Day 4 | Day 5 | Day 6 | Day 7 | Day 8 | Day 9~ Day 10 | Day 11 | Day 12~ Day 14 | Day 15  ± 2 days | Day 22  ± 3 days | Day 29  ± 3 days |
| Informed consent | ○ |  |  |  |  |  |  |  |  |  |  |  |  |  |  |
| Demography | ○ |  |  |  |  |  |  |  |  |  |  |  |  |  |  |
| Inclusion/exclusion criteria | ○ |  |  |  |  |  |  |  |  |  |  |  |  |  |  |
| Check SARS-CoV-2 results | ○ |  |  |  |  |  |  |  |  |  |  |  |  |  |  |
| Randomization^[[1]](#footnote-1)^ |  | ○ |  |  |  |  |  |  |  |  |  |  |  |  |  |
| Investigational Product Administration |  | ○ | ○ | ○ | ○ | ○ | ○ | ○ | ○ | ○ | ○ | ○ |  |  |  |
| Vital sign^[[2]](#footnote-2)^ | ○ | ○ | ○ | ○ | ○ | ○ | ○ | ○ | ○ | ○ | ○ | ○ | ○ |  | ○ |
| Clinical laboratory test^[[3]](#footnote-3)^ | ○ | ○ |  | ○ |  | ○ |  |  | ○ |  | ○ |  |  |  | ○ |
| Physical examination | ○ |  |  |  |  |  |  |  |  |  |  |  |  |  | ○ |
| 8-ordinal scale^[[4]](#footnote-4)^ |  | ○ | ○ | ○ | ○ | ○ | ○ | ○ | ○ | ○ | ○ | ○ | ○ | ○ | ○ |
| Severity classification for COVID-19^[[5]](#footnote-5)^ |  | ○ | ○ | ○ | ○ | ○ | ○ | ○ | ○ | ○ | ○ | ○ | ○ | ○ | ○ |
| National Early Warning Score^[[6]](#footnote-6)^ |  | ○ | ○ | ○ | ○ | ○ | ○ | ○ | ○ | ○ | ○ | ○ | ○ | ○ | ○ |
| Adverse events monitoring |  | ○ | ○ | ○ | ○ | ○ | ○ | ○ | ○ | ○ | ○ | ○ | ○ | ○ | ○ |
| Concomitant medication monitoring | ○ | ○ | ○ | ○ | ○ | ○ | ○ | ○ | ○ | ○ | ○ | ○ | ○ | ○ | ○ |

1. On Day 1, each patient is stratified according to the presence or absence of SARS-CoV-2 vaccination, the institution conducting the clinical trial, and the severity at the time of randomization, and randomized to either the investigational drug administration group or the placebo administration group. [↑](#footnote-ref-1)
2. Vital signs are evaluated daily during the treatment period (Day 1 ~ Day 14) and at follow-up (15d and 29d), and eardrum temperature (C°), respiratory rate (breathing per minute), systolic and diastolic blood pressure (mmHg), and pulse rate (bpm) are measured. If the patient cannot visit the clinical trial site at the time of follow-up, evaluation is not performed. [↑](#footnote-ref-2)
3. Hematology tests, blood chemistry tests, blood coagulation tests, and urinalysis are performed at the screening period, treatment period (before administration on Day 1, Day 3, Day 5, Day 8, and Day 11), and at the last follow-up period (Day 29). During the screening period, urine drug test, serology test, and pregnancy test are additionally performed to confirm selection exclusion criteria. [↑](#footnote-ref-3)
4. Ordinal scale evaluation of clinical signs is performed every day during the treatment period (Day 1 ~ Day 14) and is performed at the follow-up periods (Day 15, Day 22 and Day 29). If a patient is unable to visit the clinical trial site during the follow-up period, an interview can be made by phone. [↑](#footnote-ref-4)
5. After administration of the investigational product, severity check for COVID-19 infection is performed daily during the treatment period (Day 1 ~ Day 14) and at the follow-up periods (Day 15, Day 22 and Day 29). If a patient cannot visit the clinical trial site during the follow-up period, an interview can be done by phone. [↑](#footnote-ref-5)
6. National Early Warning Score evaluation is performed daily during the treatment period (Day 1 ~ Day 14) and at follow-up periods (Day 15, Day 22, Day 29). If the patient cannot visit the clinical trial site at the time of follow-up, evaluation is not performed. [↑](#footnote-ref-6)
